# Ionic Liquids as Solvents for Rhodium and Platinum Catalysts Used in Hydrosilylation Reaction

**DOI:** 10.3390/molecules21091115

**Published:** 2016-08-24

**Authors:** Witold Zielinski, Rafal Kukawka, Hieronim Maciejewski, Marcin Smiglak

**Affiliations:** 1 Poznan Science and Technology Park, Adam Mickiewicz University Foundation, 46 Rubież ST., 61-612 Poznań, Poland; witar6@gmail.com (W.Z.); kukawka.rafal@gmail.com (R.K.); maciejm@amu.edu.pl (H.M.); 2 Faculty of Chemistry, Adam Mickiewicz University, Umultowska 89b, 61-614 Poznań, Poland

**Keywords:** ionic liquids, catalysis, hydrosilylation reaction

## Abstract

A group of imidazolium and pyridinium based ionic liquids has been synthetized, and their ability to dissolve and activate the catalysts used in hydrosilylation reaction of 1-octane and 1,1,1,3,5,5,5-heptamethyltrisiloxane was investigated. An organometallic catalyst as well as inorganic complexes of platinum and rhodium dissolved in ionic liquids were used, forming liquid solutions not miscible with the substrates or with the products of the reaction. The results show that application of such a simple biphasic catalytic system enables reuse of ionic liquid phase with catalysts in multiple reaction cycles reducing the costs and decreasing the amount of catalyst needed per mole of product.

## 1. Introduction

**Figure 1:**
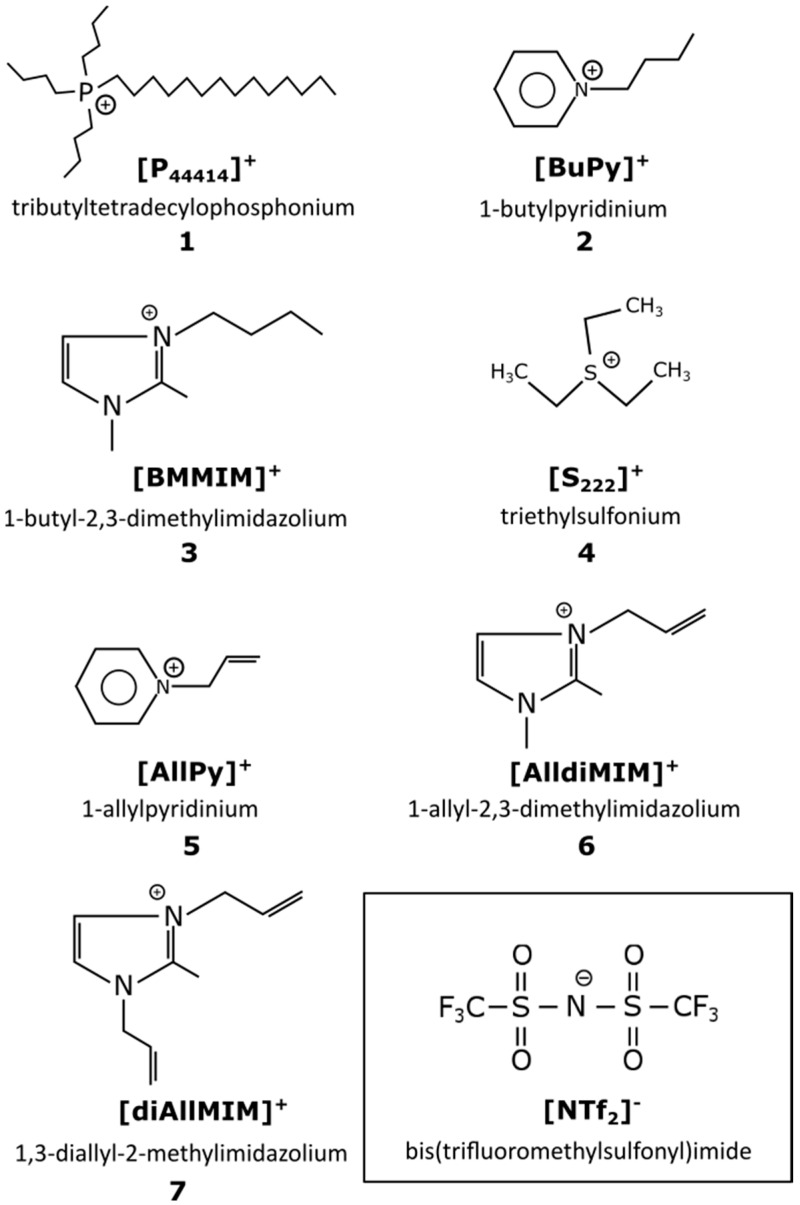
Schematic representation of ionic liquids used as solvent for catalyst in hydrosilylation reaction (1–7). All ILs have the same anion: bis(trifluoromethylsulfonyl)imide—[NTf_2_]^−^ (highlighted by a frame).

Recently, there has been major interest in more “green” and environmentally-friendly approaches to industrial processes and technologies. Very helpful in obtaining such goals can be compounds known as ionic liquids (ILs) [1]. The most commonly used definition of ILs refers to their melting point lying below 100 °C, which means, that they are salts melted at room temperature (or below 100 °C) composed of organic or inorganic cations and anions. From these ionic structures emerge interesting properties such as: low viscosity; low vapor pressure [2]; and good dissolution in many inorganic, organic and polymeric compounds [3]. Huge interest in IL chemistry, and intensification of research on ILs in the last decade is reflected in a growing number of publications referring to IL properties, synthesis and applications [1,4,5,6] starting with their solvent properties in organic synthesis [7] as well as use in energy storage materials [8], medicine [9], catalysis [10], separation science [11] or crystallography [12] to name only a few. However, the most useful feature of ILs is their tunability of properties possible through chemical modifications of cations or anions and hence adjustment of the intermolecular and interionic interactions [12]. The combinations of cations and anions composing ILs and applications for them are growing on a daily basis. The cations are generally bulky, asymmetric ammonium or phosphonium salts, or heteroaromatics, with low symmetry, weak intermolecular interactions and low charge densities. Typically, the anions are described as non-coordinating, inorganic ions and include [PF_6_]^−^, [BF_4_]^−^, [CF_3_SO_3_]^−^ or [(CF_3_SO_2_)_2_N]^−^, although, more recently, organic anions (e.g., [RCO_2_]^−^) have also been introduced [13]. The physical and chemical properties of the ionic liquid, including their melting points, are dependent on both the nature of the cation and the anion. A schematic representation of ILs used in our study is presented in Figure 1.

Solvent effect on reactions is one of the most interesting issues related to research on ILs [14]. The ILs have been used successfully as a media for many reactions [15], for example carbon–carbon bond forming reactions (Knoevenagel, Michael Aldol, Biginelli, Heck reactions), substitution, elimination, addition (i.e., nucleophilic, electrophilic aromatic substitution, esterification, Diels–Alder) acid catalyzed reactions and transition-metal catalyzed reactions i.e., hydrogenation, oxidation or carbonylation. There are also many possibilities of IL application in catalysis [16] i.e., as the catalyst itself, as a co-catalyst, as a catalyst activator, as the source of a new ligand for a catalytic metal center, or as a solvent for the reaction. There are many examples of catalytic processes involving ILs and one of them is the hydrosilylation reaction in which ILs immobilize platinum [17] or rhodium [18] catalysts used during biphasic reactions in batch. There have been several comprehensive papers regarding hydrosilylation reaction carried in the presence of ILs [19,20,21], but articles usually focus on a narrow scope of catalyst/IL systems, usually investigating in-depth one particular catalyst. As it is reported by Hoffman et al. [22], most suitable for hydrosilylation reaction are salts with the [NTf_2_]^−^ anion. Thus, during our study, we have focused primarily on the investigation of various catalysts and their performance in different ILs, specifically containing [NTf_2_]^−^ anion, thus being able to investigate the influence of chemical modification of the cation on the catalyst activity. Utilization of a biphasic system allows for reducing the amount of catalyst used during the process by reuse of IL-phase in subsequent cycles. This simple method results also in decreased contamination of product with catalyst. This aspect is especially important since hydrosilylation reaction products are very commonly used in the cosmetic and food industry [23].

**Figure 2:**
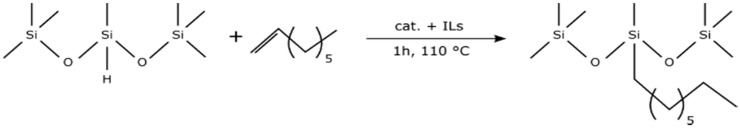
Hydrosilylation reaction of 1-octene and 1,1,1,3,5,5,5-heptamethyltrisiloxane.

Due to their unique properties, organosilicon compounds are widely used and have become indispensable in our daily life. Through their dual reactivity, organosilanes serve as bridges between inorganic or organic substrates (such as minerals, fillers, metals and cellulose) and organic/polymeric matrices (such as rubber, thermoplastics or thermosets) and, hence, can dramatically improve adhesion between them [24]. Organosilicons consist mainly of Si–C bonds that do not exist naturally and have to be synthesized via hydrosilylation reaction [25], one of the most convenient and widely used methods of forming Si–C bonds. It involves the addition of the Si–H bond in hydrosilane across an unsaturated hydrocarbon such as an alkene or alkyne (Figure 2). For such a reaction, a transition-metal catalyst is required [26]. Among many transition-metal catalysts reported to date, platinum catalysts such as Speier’s catalyst and Karstedt’s catalyst are known to be very effective and are often used commercially [27]. However, we know that the use of precious metals in catalysis is not always desired and the used amount should be as little as possible. The relatively high cost, environmental concerns, and uncertainty of the long-term supply of precious metals have inspired the search for equivalent or superior catalysts, or improved synthetic methods that allow for minimizing the amount of used catalysts.

Hence, in our study, we have proposed a more economic approach to catalytical hydrosilylation reaction with the use of ILs. This very simple concept involves two immiscible liquids creating a biphasic system, where one phase constitutes IL with a dissolved catalyst, and another is composed of substrates and after time products. This approach allows for separation of both phases and reuse of the IL phase with catalysts. In some cases, the IL phase was used up to more than five cycles before catalytic properties were lost and a new IL phase had to be prepared. This approach in contrast to the traditional approach allows saving money spent on catalysts and enables construction of closed cycle reaction systems. In order to isolate best pairs of IL/catalysts for hydrosilylation reactions, we have investigated six catalysts and seven ILs.

## 2. Results

During this study, 42 possible combinations of ILs and catalysts were investigated (Table 1). The reaction conversion and yield were controlled by gas chromatography and obtained results allowed the emergence of best IL/catalyst pairs. In our opinion, three out of seven tested IL systems have proven to be useful in five subsequent cycles with yields maintained at a level of around 80%.

**Figure 3:**
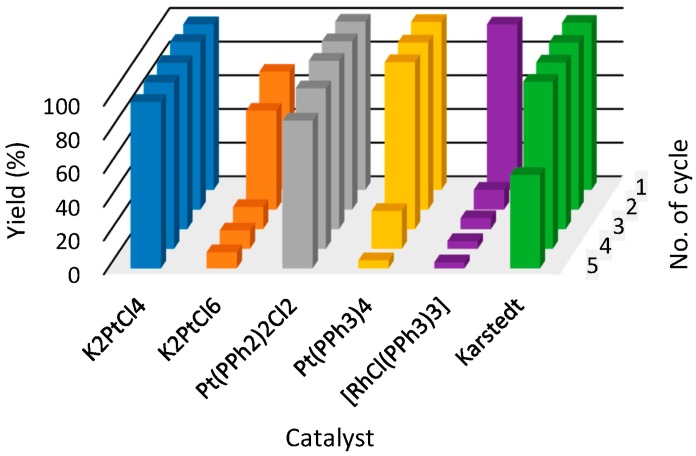
The yields of hydrosilylation reaction carried out in [P_44414_][NTf_2_] (**1**) (tributyltetradecylphosphonium bis(trifluoromethylsulfonyl)imide) in subsequent cycles. Colors refer to different catalysts used in hydrosilylation reaction.

**Figure 4:**
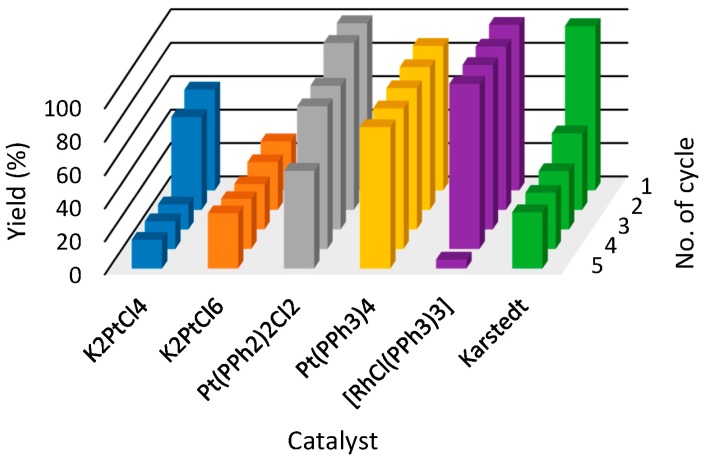
The yields of hydrosilylation reaction carried out in [BMMIM][NTf_2_] (**3**) (1-butyl-2,3-dimethylimidazolium bis(trifluoromethylsulfonyl)imide) in subsequent cycles. Colors refer to different catalysts used in hydrosilylation reaction.

**Figure 5:**
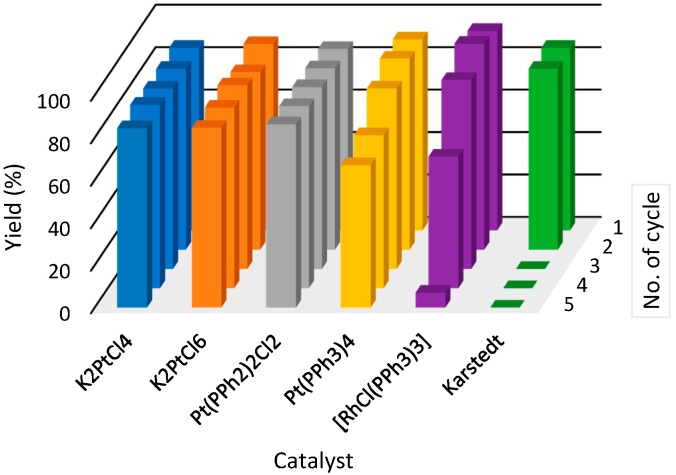
The yields of hydrosilylation reaction carried out in [S_222_](NTf_2_) (**4**) (triethylsulfonium bis(trifluoromethylsulfonyl)imide) in subsequent cycles. Colors refer to different catalysts used in hydrosilylation reaction.

Results for the three most promising ILs: [P_44414_][NTf_2_] (**1**), [BMMIM][NTf_2_] (**3**) and [S_222_][NTf_2_] (**4**), are shown in the form of 3D bar graphs in Figure 3, Figure 4 and Figure 5, respectively. [P_44414_][NTf_2_] (**1**) IL system shows satisfying yields close to 100% throughout the whole five reaction cycles for catalysts such as [RhCl(PPh_3_)_3_], Pt(PPh_3_)_2_Cl_2_ and Karstedt catalyst (100% yield up to forth cycle). A promising catalyst which shows a yield close to 100% throughout the first three reaction cycles was Pt(PPh_3_)_3_; however, unfortunately, after a fourth cycle, 22% yield was observed. Platinum (IV) catalyst shows moderate yields at a level of 70% after the first cycle and 4% after a third cycle. A less effective catalyst is also K_2_PtCl_6_, in which, after a second reaction cycle, a 13% yield was observed. The least effective catalyst for IL (**1**) is platinum catalyst, K_2_PtCl_4_, for which a major drop in yield was observed after the first reaction cycle.

The second IL system, in which promising results were obtained, is [BMMIM][NTf_2_] (**3**). This IL, when used with Pt(PPh_2_)_2_Cl_2_ and Pt(PPh_3_)_3_ catalysts, showed best yields close to 100% (decreased to 60% after last reaction cycle) and around 85%, respectively. In the case of rhodium catalyst, a 100% yield was sustained up to a fourth cycle, when a major drop from 99% to 5% was then observed. In the case of K_2_PtCl_4_, K_2_PtCl_6_ and Karstedt catalysts, the yields after the second cycle have reached unacceptable values.

In the case of [S_222_][NTf_2_] (**4**) yields did not reach a level of 100%, but they remained at a satisfactory level of more than 80% yield throughout whole five cycles for almost all of the catalysts used. Only in the case of three catalysts—[RhCl(PPh_3_)_3_], Pt(PPh_3_)_4_ and Karstedt catalysts—yields were dropping after a fourth cycle to unacceptable levels of less than 70%. In the case of Karstedt catalyst, after a second cycle, products of hydrosilylation reaction were not observed. It seems that the most suitable IL/catalyst pairs for hydrosilylation reaction with the [S_222_][NTf_2_] (**4**) IL are platinum catalysts: K_2_PtCl_4_, K_2_PtCl_6_, Pt(PPh_3_)_2_Cl_2_, in which yields were maintained at a level of around 80%.

The least effective ILs systems are [BuPy][NTf_2_] (**2**) [diAllMIM][NTf_2_] (**6**) and [AlldiMIM][NTf_2_] (**7**), which yields, in almost every example, a range between 0 and 30%. The border line case is [AllPy][NTf_2_] (**5**) IL system, in which for almost all catalysts, good yields are observed after first and second cycles but after a fifth one, almost no product of hydrosilylation reaction is observed.

## 3. Discussion

There are more than 100 examples of utilization of ILs in different types of organic reactions [28]. It is also known that ILs can serve as catalysts, catalysts immobilizers or initiators in many chemical processes [29]. These proved properties were inspiration for our work on using ILs in hydrosilylation reaction. Utilization of ILs for this process was proposed because of several reasons:

- hydrosilylation reaction products are often used in cosmetics in which presence of heavy metals (catalyst residue) is unacceptable; thus, utilization of ILs allows for better separation of phases containing catalysts and reaction products,
- thermal stability of ILs allows for better control over reaction conditions,
- reusable IL phase containing catalyst in subsequent cycles allows for reduction of utilized catalyst and hence decreases the cost of the process,
- a simple biphasic system allows for construction of closed cycle reaction systems that increase productivity/time ratio, which also leads to reduction of overall reaction cost,
- hydrosilylation reaction of 1,1,1,3,5,5,5-hepthamethyltrisiloxane and 1-octene is a model system on which catalytical properties of such biphasic systems can be tested and applied for other compounds.

Imidazolium and pyridinium based ILs have been selected as a most suitable group of ILs for investigation in hydrosilylation reaction due to a few major factors: (i) proved good solubility of many catalysts in ILs containing these cations; (ii) high chemical stability of imidazolium and pyridinium IL systems; (iii) proven high conversion rates of the reactions carried out using biphasic systems with catalysts being dissolved in IL phase; and (iv) often immiscibility of the reaction products in IL phase and lack of washing out of the catalyst from the IL phase [19,20,22]. Furthermore, those ILs are also one of the most commonly used ILs in organic synthesis [2], and they are relatively cheap and easy to handle and synthetize.

The project aim was to investigate the influence of IL systems with catalysts dissolved within them on yields of hydrosilylation reactions and to determine how modifications of side chains, connected with imidazolium or pyridine based ILs (or lack of unsaturated chains), influence the yields of hydrosilylation reactions. As an anion for all ILs utilized during this study, bis(trifluoromethylsulfonyl)imide anion ([NTf_2_]^−^) was selected. [NTf_2_]^−^ anion is often used to form ILs known for their chemical inertness and hydrophobic properties and hence was most suitable for this study [22]. This second feature of [NTf_2_]^−^ is also crucial since water is often responsible for catalyst deactivation during hydrosilylation reactions. [NTf_2_]^−^ anion is also non-coordinating and a passive anion that guarantees no interactions between anion and catalyst.

The use of IL systems, as proposed in the described studies, guarantees recovery of the IL solvent due to its property, insolubility in substrates nor products of the reaction. Moreover, as seen from the results of tests performed with recycled IL/catalyst systems, for most of the reactions performed, it is possible to recover the catalyst from the reaction mixture and reuse it in the consecutives cycles. With mass control over IL phase, it is also possible to determinate whenever IL phase is flushed out of reaction systems. Unfortunately, it is not possible to apply the inductively coupled plasma (ICP) technique for quantitative determination of catalysts present within ILs, since amounts of used catalysts are close to the ICP detection limits.

This study clearly shows that utilization of a biphasic IL/catalyst system for hydrosilylation reaction can majorly decrease amounts of used catalyst and hence costs of the process. The most efficient IL systems for hydrosilylation reaction were [P_44414_][[NTf_2_] (**1**)/[RhCl(PPh_3_)_3_] and [P_44414_][NTf_2_] (**1**)/Pt(PPh_3_)_4_, for which yields after a fifth cycle were maintained at a level of more than 80%.

On the other hand, we observed the drastic loss of catalyst activity after the first reaction cycle in the case of many pair catalysts/ILs investigated; however, the most apparent one is related to the behavior of [RhCl(PPh_3_)_3_], which in three out of seven tested IL systems shows an immediate drop of the catalyst activity when being recycled for the first time. In another system (with IL [diAllMIM][NTf_2_]) (**7**), this catalyst shows no activity at all (which was observed for all other catalysts as well), and in other two examples with salts previously investigated by our group, ([BMMIM][NTf_2_] (**3**) and [S_222_][NTf_2_]) (**4**), high yields of conversion were observed even after a third cycle.

Moreover, we have noted that the introduction of the unsaturated double bond to the structure of the IL solvent leads to almost immediate deactivation of the catalysts (any of the catalysts). We speculate that the presence of the double bond leads to its strong coordination to the metal complex, thus making it unreactive or a concurrent hydrosilylation reaction between HMTS and C=C double bond in the IL. For instance, when comparing the catalyst activity in the systems containing IL [BMMIM][NTf_2_] (**3**) vs. [AlldiMIM][NTf_2_] (**6**) and [diAllMIM][NTf_2_] (**7**), where the only difference between the ILs is the presence of the double bond on the alkyl chain in [AlldiMIM][NTf_2_] (**6**) and [diAllMIM][NTf_2_] (**7**), it can be clearly seen that the catalysts activity (for all of the catalysts) drops drastically even at the first reaction cycle, whereas in the case of [BMMIM][NTf_2_] (**3**) catalyst, activity is maintained for some of the catalysts at a moderate level over a few reaction cycles. Similar behavior can be observed when comparing the ILs [AllPy][NTf_2_] (**5**) (containing unsaturated C=C bond in the alkyl chain) and [BuPy][NTf_2_] (**2**). It appears that the presence of double bond in side chain lowers the yields of hydrosilylation reactions. It might be caused by competitive reaction of silanes with double bonds of an IL system. Hence, most effective ILs systems are those without double bonds despite their potential stabilization effect on catalysts.

## 4. Materials and Methods

Potassium tetrachloroplatinate, K_2_PtCl_4_ (98%, Sigma Aldrich, Saint Louis, MO, USA), potassium hexachloroplatinate, K_2_PtCl_6_ (98%, Sigma Aldrich), bis(triphenylphosphine)platinum chloride, Pt(PPh_3_)_2_Cl_2_ (98%, Sigma Aldrich), tetrakis(triphenylphosphine)platinum(0), Pt(PPh_3_)_4_ (98%, Sigma Aldrich), tris(triphenylphosphine)rhodium (I) chloride, [RhCl(PPh_3_)_3_] also known as Wilkinson’s catalyst (98%, Sigma Aldrich), Karstedt catalyst—platinum(0)-1,3-divinyl-1,1,3,3-tetramethyldisiloxane complex (2.2% solution in xylene, Sigma Aldrich), pyridine (Sigma Aldrich), 2-methylimidazole (Sigma Aldrich), 1,2-dimethylimidazole (Sigma Aldrich), tributyltetradecylphosphonium chloride [P_44414_][Cl] (IoLiTec—Ionic Liquids Technologies GmbH, Helibronn, Germany), 1-butyl-2,3-dimethylimidazolium chloride [BMMIM][Cl] (IoLiTec), triethylsulfonium bis(trifluoromethylsulfonyl)imide [S_222_][NTf_2_] (**4**) (IoLiTec), bis(trifluoromethylsulfonyl)imide lithium salt, LiNTf_2_ (IoLiTec) were used as received.

### 4.1. Synthesis of Ionic Liquids

#### 4.1.1. Synthesis of 1,3-Diallyl-2-methylimidazolium Chloride [diAllMIM][Cl]

To 1 equivalent of 2-methylimidazole in toluene an equivalent of NaOH was added (dissolved in water). The reaction was carried out under reflux. After 3 h, the reaction mixture was cooled to 50 °C and allyl chloride (1 molar equivalent) was added dropwise. The reaction was intensive stirred for the next 2 days. Subsequently, after phase separation, the toluene phase was evaporated and the product was used in the next reaction step. Obtained 1-allyl-2-methylimidazole was dissolved in acetonitrile, to which 1.1 equivalent of allyl chloride was added. Reaction was carried out for one week at 50 °C (not in reflux) in order to avoid unwanted byproducts. Subsequently, acetonitrile was evaporated. The product was washed twice with diethyl ether and dried under high vacuum (1 mbar) in 60 °C for 24 h. The structure of obtained salts were confirmed by ^1^H-NMR analysis.

#### 4.1.2. Synthesis of 1-Butylpyridinium Bromide [BuPy][Br], 1-Allylpyridinium Chloride [AllPy][Cl], 1-Allyl-2,3-dimethylimidazolium Chloride [AlldiMIM][Cl] Performed by Alkylation Reaction Using General Procedure

One molar equivalent of pyridine, 1,2-dimethylimidazole was dissolved in acetonitrile. After complete dissolution of the substrate, 1 molar equivalent of alkyl halide or allyl chloride was added dropwise to reaction mixture. The reaction was carried out for one week at 50 °C (not in reflux) in order to avoid unwanted byproducts. Subsequently, acetonitrile was evaporated. The product was washed twice with diethyl ether and dried under high vacuum (1 mbar) in 60 °C for 24 h. The structure of obtained salts were confirmed by ^1^H-NMR analysis.

#### 4.1.3. Synthesis of Ionic Liquids with Bis(trifluoromethylsulfonyl)imide [NTf_2_]^−^

Tributyltetradecylphosphonium bis(trifluoromethylsulfonyl)imide [P_44414_][NTf_2_] (**1**); 1-butyl-2,3-dimethylimidazolium bis(trifluoromethylsulfonyl)imide [BMMIM][NTf_2_] (**3**); 1-butylpyridinium bis(trifluoromethylsulfonyl)imide [BuPy][NTf_2_] (**2**); 1-allylpyridinium bis(trifluoromethylsulfonyl)imide [AllPy][NTf_2_] (**5**); 1-allyl-2,3-dimethylimidazolium bis(trifluoromethylsulfonyl)imide [AlldiMIM][NTf_2_] (**7**) and 1,3-diallyl-2-methylimiidazolium bis(trifluoromethylsulfonyl)imide [diAllMIM][NTf_2_] (**6**) were synthesized by metathesis reaction using the general procedure:

Pyridinium or imidazolium halide (bromide or chloride) was dissolved in water and it was transferred to a separator funnel with dichloromethane. Subsequently, 1.1 molar equivalent of LiNTf_2_ (80% solution in water) was added and a separator funnel was shaken vigorously. After phase separation, the organic phase was washed twice with water, once with 5% water solution of LiNTf_2_, and this procedure was repeated until water phase was completely free of halide anion. To confirm absence of chloride anion, acidic solution of silver nitrate was added to a sample of water phase. No precipitate of silver chloride indicated an absence of chloride anion. Subsequently, the organic phase was separated, evaporated and dried under high vacuum (1 mbar) in 60 °C for 24 h. The structure of obtained salts were confirmed by ^1^H-NMR analysis.

The ^1^H-NMR analysis was applied for all ILs used during this study and the results are listed below:

*Tributyltetradecylphosphonium bis(trifluoromethylsulfonyl)imide [P_44414_][NTf_2_]* (**1**). ^1^H-NMR (300 MHz, DMSO-*d*_6_, 25 °C, TMS): δ/ppm = 2.16 (8H, t, P–CH_2_–), 1.21–1.47 (36H, m, P–alkyl–), 0.92 (9H, t, *J* = 6.79 Hz, –CH_3_), 0.86 (3H, t, *J* = 6.89 Hz, –CH_3_).

*1-Butylpyridinium bis(trifluoromethylsulfonyl)imide [BuPy][NTf_2_]* (**2**). ^1^H-NMR (300 MHz, DMSO-*d*_6_, 25 °C, TMS): δ/ppm = 9.08 (2H, d, *J* = 5.57 Hz, Py–H), 8.59 (1H, t, *J* = 7.86 Hz, Py–H), 8.14 (2H, t, *J* = 7.14 Hz, Py–H), 4.61 (2H, t, *J* = 7.36 Hz, NCH_2_–), 1.91 (2H, p, *J* = 7.14 Hz, NCH_2_CH_2_–), 1.29 (2H, m, –CH_2_–CH_3_ 0.92 (3H, t, *J* = 7.14 Hz, –CH_3_).

*1-Butyl-2,3-dimethylimidazolium bis(trifluoromethylsulfonyl)imide [BMMIM][NTf_2_]* (**3**). ^1^H-NMR (300 MHz, DMSO-*d*_6_, 25 °C, TMS): δ/ppm = 7.61 (1H, s, IM–H), 7.60 (1H, s, IM–H), 4.10 (2H, t, *J* = 7.43 Hz, NCH_2_–), 3.75 (3H, s, NCH_3_), 2.58 (3H, s, IM–CH_3_), 1.70 (2H, p, *J* = 7.25 Hz, NCH_2_–CH_2_–), 1.30 (2H, m, –CH_2_–CH_3_ 0.91 (3H, t, *J* = 7.43 Hz, –CH_3_).

*Triethylsulfonium bis(trifluoromethylsulfonyl)imide [S_222_][NTf_2_]* (**4**) ^1^H-NMR (300 MHz, DMSO-*d*_6_, 25 °C, TMS): δ/ppm = 3.21 (6H, q, *J* = 7.62 Hz, SCH_2_–), 1.35 (9H, t, *J* = 7.62 Hz, –CH_3_).

*1-Allylpyridinium bis(trifluoromethylsulfonyl)imide [AllPy][NTf_2_]* (**5**) ^1^H-NMR (300 MHz, DMSO-*d*_6_, 25 °C, TMS): δ/ppm = 9.02 (2H, d, *J* = 5.40 Hz, Py–H), 8.62 (1H, t, *J* = 7.89 Hz, Py–H), 8.16 (2H, t, *J* = 6.83 Hz, Py–H), 6.16 (1H, m, –CH=CH_2_), 5.45 (1H, d, *J* = 10.10 Hz, –CH=CH_2_), 5.41 (1H, d, *J* = 16.89 Hz, –CH=CH_2_), 5.29 (2H, d, *J* = 6.34 Hz, NCH_2_–).

*1,3-Diallyl-2-methyliidazolium bis(trifluoromethylsulfonyl)imide [diAllMIM][NTf_2_]* (**6**) ^1^H-NMR (300 MHz, DMSO-*d*_6_, 25 °C, TMS): δ/ppm = 7.64 (2H, s, IM–H), 6.00 (2H, m, *J* = 5.43 Hz, –CH=CH_2_), 5.34 (2H, d, *J* = 10.85 Hz, –CH=CH_2_), 5.18 (2H, d, *J* = 16.75 Hz, –CH=CH_2_), 4.83 (4H, d, *J* = 5.43 Hz, NCH_2_–), 2.54 (3H, s, IM–CH_3_).

*1-Allyl-2,3-dimethylimidazolium bis(trifluoromethylsulfonyl)imide [AlldiMIM][NTf_2_]* (**7**) ^1^H-NMR (300 MHz, DMSO-*d*_6_, 25 °C, TMS): δ/ppm = 7.62 (1H, s, IM–H), 7.57 (1H, s, IM–H), 5.98 (1H, m, –CH=CH_2_), 5.31 (1H, d, *J* = 10.32 Hz, –CH=CH_2_), 5.14 (1H, d, *J* = 17.25 Hz, –CH=CH_2_), 4.80 (2H, d, *J* = 5.47 Hz, NCH_2_–), 3.76 (3H, s, NCH_3_), 2.54 (3H, s, IM–CH_3_).

### 4.2. Catalyst Activity Experiments

Reactions were performed in small scale (4 mL) glass reactors in which an equivalent quantity of 1 × 10^−4^ mole of catalyst metal per mole of Si–H bond was placed. Then, the reactor was filled with IL in a quantity of 10% of total reaction volume (0.158 mL). In order to dissolve and activate the catalyst, the reactor was stirred and heated for 20 min at a temperature of 110 °C. After cooling to room temperature, a mixture of 1-octene (0.578 mL, 0.0037 mol) and 1,1,1,3,5,5,5-heptatrimethylotrisiloksane HMTS (1 mL, 0.0037 mol) was added. As an internal standard, 1-decane was used (0.158 mL). The reactions were carried out under reflux condenser at the temperature of 110 °C with stirring. After 1 h, the solution was cooled to room temperature and phases were separated. The upper phase containing a product of hydrosilylation reaction was separated and investigated using gas chromatography; bottom phase containing IL, with catalyst, was used in further reaction cycles with a new mixture of reaction substrates. Gas chromatography analyses were carried out on a Varian 3800 Agilent Technologies chromatograph (Santa Clara, CA, USA) (equipped with a DB-1, 30 m capillary column and thermal conductivity detector (TCD), temperature program: 60 °C (3 min) heating 10 °C min^−1^ till 300 °C (10 min)).

## 5. Conclusions

A new approach to hydrosilylation reactions with the use of a biphasic system of IL with a dissolved catalyst and a separate phase with reactants/products was under investigation. The ability of this simple system to reduce the amount of catalysts used for hydrosilylation reactions, due to their multiple usages, was proved to be useful. Obtained results clearly show that using IL in this reaction has many advantages such as reduction of catalysts used due to reuse of IL-phase (with catalyst dissolved) in new reaction cycles and hence possible reduction of costs at industrial scale. This two-phase reaction system also allows separation of catalysts from reaction products (no contamination with heavy metals) that is especially important since organofunctional silanes are widely used i.e., in dermatological products. Our screening research highlighted the most promising ILs as candidates for study in flow-through microreactor systems [30], which we are going to conduct in the near future. This, on the other hand, can lead to determination of the most efficient conditions for hydrosilylation reaction in closed circuits.

## Figures and Tables

**Table 1:** Yields of hydrosilylation reaction in subsequent cycles for biphasic systems of ionic liquids and catalysts used.

Ionic Liquid	Catalyst	Yields in Subsequent Cycles (%) ^1^				
		1	2	3	4	5
**[P_44414_][NTf_2_] 1**	K_2_PtCl_4_	98.34	11.73	6.56	4.53	3.57
	K_2_PtCl_6_	70.26	58.65	13.09	10.87	9.56
	Pt(PPh_3_)_2_Cl_2_	100.00	100.00	100.00	95.25	87.74
	Pt(PPh_3_)_4_	99.82	99.22	99.13	22.40	4.77
	[RhCl(PPh_3_)_3_]	98.45	99.60	98.68	98.71	98.71
	Karstedt	99.27	99.12	98.95	99.19	55.41
**[BuPy][NTf_2_] 2**	K_2_PtCl_4_	85.68	2.25	0.39	0.75	3.92
	K_2_PtCl_6_	86.05	3.42	5.47	10.74	13.58
	Pt(PPh_3_)_2_Cl_2_	84.22	2.30	5.23	5.68	6.38
	Pt(PPh_3_)_4_	16.12	14.79	13.14	11.46	11.52
	[RhCl(PPh_3_)_3_]	100.00	12.22	9.56	2.58	8.91
	Karstedt	100.00	80.09	16.02	17.37	9.44
**[BMMIM][NTf_2_] 3**	K_2_PtCl_4_	60.26	55.68	15.15	16.75	17.40
	K_2_PtCl_6_	29.35	28.69	27.45	30.26	33.48
	Pt(PPh_3_)_2_Cl_2_	100.00	100.00	86.24	85.84	59.07
	Pt(PPh_3_)_4_	86.39	85.46	84.81	84.52	85.18
	[RhCl(PPh_3_)_3_]	98.99	97.82	98.71	99.27	5.44
	Karstedt	98.30	45.89	35.15	34.05	34.12
**[S_222_][NTf_2_] 4**	K_2_PtCl_4_	86.03	85.17	84.93	86.35	84.41
	K_2_PtCl_6_	87.80	83.62	86.47	85.03	84.57
	Pt(PPh_3_)_2_Cl_2_	85.52	85.43	85.66	85.77	86.30
	Pt(PPh_3_)_4_	90.00	90.00	85.00	72.00	67.00
	[RhCl(PPh_3_)_3_]	94.00	97.00	89.00	62.00	7.00
	Karstedt	86.03	85.17	0.00	0.00	0.00
**[AllPy][NTf_2_] 5**	K_2_PtCl_4_	58.00	47.00	14.00	3.00	1.00
	K_2_PtCl_6_	91.00	66.00	31.00	10.00	1.00
	Pt(PPh_2_)_2_Cl_2_	47.00	32.00	23.00	14.00	9.00
	Pt(PPh_3_)_4_	39.00	22.00	13.00	3.00	1.00
	[RhCl(PPh_3_)_3_]	88.00	0.00	0.00	x	x
	Karstedt	97.15	93.70	57.58	6.08	0.00
**[diAllMIM][NTf_2_] 6**	K_2_PtCl_4_	1.00	0.00	0.00	x	x
	K_2_PtCl_6_	0.00	0.00	0.00	x	x
	Pt(PPh_3_)_2_Cl_2_	15.00	11.00	8.00	9.00	2.00
	Pt(PPh_3_)_4_	3.00	3.00	3.00	0.00	0.00
	[RhCl(PPh_3_)_3_]	0.00	0.00	0.00	0.00	0.00
	Karstedt	16.00	9.00	6.00	2.00	0.00
**[AlldiMIM][NTf_2_] 7**	K_2_PtCl_4_	10.00	8.00	2.00	0.00	0.00
	K_2_PtCl_6_	2.00	5.00	3.00	0.00	0.00
	Pt(PPh_3_)_2_Cl_2_	35.00	27.00	20.00	14.00	11.00
	Pt(PPh_3_)_4_	30.00	25.00	19.00	8.00	0.00
	[RhCl(PPh_3_)_3_]	78.00	18.00	2.00	0.00	0.00
	Karstedt	30.00	15.00	8.00	3.00	0.00

^1^ Yields color code: green >90%; blue 70%–90%; orange 50%–70%; yellow 30%–50%; red 0%–30%; [P_44414_][NTf_2_] tributyltetradecylphosphonium bis(trifluoromethylsulfonyl)imide; [BuPy][NTf_2_] 1-butylpyridinium bis(trifluoromethylsulfonyl)imide; [BMMIM][NTf_2_] 1-butyl-2,3-dimethylimidazolium bis(trifluoromethylsulfonyl)imide; [S_222_][NTf_2_] triethylsulfonium bis(trifluoromethylsulfonyl)imide; [AllPy][NTf_2_] 1-allylpyridinium bis(trifluoromethylsulfonyl)imide; [diAllMIM][NTf_2_] 1,3-diallyl-2-methylimiidazolium bis(trifluoromethylsulfonyl)imide; [AlldiMIM][NTf_2_] 1-allyl-2,3-dimethylimidazolium bis(trifluoromethylsulfonyl)imide.
