# Correction: Zielinski, W., et al. Ionic Liquids as Solvents for Rhodium and Platinum Catalysts Used in Hydrosilylation Reaction. *Molecules* 2016, *21*, 1115

**DOI:** 10.3390/molecules22071203

**Published:** 2017-07-18

**Authors:** Witold Zielinski, Rafal Kukawka, Hieronim Maciejewski, Marcin Smiglak

**Affiliations:** 1 Poznan Science and Technology Park, Adam Mickiewicz University Foundation, 46 Rubież ST., 61-612 Poznań, Poland; witar6@gmail.com (W.Z.); kukawka.rafal@gmail.com (R.K.); maciejm@amu.edu.pl (H.M.); 2 Faculty of Chemistry, Adam Mickiewicz University, Umultowska 89b, 61-614 Poznań, Poland

The authors are sorry to report that the yield of the hydrosilylation reaction in [P_44414_][NTf_2_] (**1**) IL with [RhCl(PPh_3_)_3_] was replaced with the yield reported for [P_44414_][NTf_2_] (**1**) IL with K_2_PtCl_4_ in their published paper [1]. Due to this fact, we would like to replace Table 1 and Figure 3, and make four changes in manuscript text to correct this mistake. Due to mislabeling, the following table and figure must be replaced:

## 1. Former Table 1

**Table 1:** Yields of hydrosilylation reaction in subsequent cycles for biphasic systems of ionic liquids and catalysts used.

Ionic Liquid	Catalyst	Yields in Subsequent Cycles (%) ^1^					
		1	2		3	4	5
**[P_44414_][NTf_2_] 1**	K_2_PtCl_4_	98.45	99.60		98.68	98.71	98.71
	K_2_PtCl_6_	70.26	58.65		13.09	10.87	9.56
	Pt(PPh_2_)_2_Cl_2_	100.00	100.00		100.00	95.25	87.74
	Pt(PPh_3_)_4_	99.82	99.22		99.13	22.40	4.77
	[RhCl(PPh_3_)_3_]	98.34	11.73		6.56	4.53	3.57
	Karstedt	99.27	99.12		98.95	99.19	55.41
**[BuPy][NTf_2_] 2**	K_2_PtCl_4_	85.68	2.25		0.39	0.75	3.92
	K_2_PtCl_6_	86.05	3.42		5.47	10.74	13.58
	Pt(PPh_2_)_2_Cl_2_	84.22	2.30		5.23	5.68	6.38
	Pt(PPh_3_)_4_	16.12	14.79		13.14	11.46	11.52
	[RhCl(PPh_3_)_3_]	100.00	12.22		9.56	2.58	8.91
	Karstedt	100.00	80.09		16.02	17.37	9.44
**[BMMIM][NTf_2_] 3**	K_2_PtCl_4_	60.26	55.68		15.15	16.75	17.40
	K_2_PtCl_6_	29.35	28.69		27.45	30.26	33.48
	Pt(PPh_2_)_2_Cl_2_	100.00	100.00		86.24	85.84	59.07
	Pt(PPh_3_)_4_	86.39	85.46		84.81	84.52	85.18
	[RhCl(PPh_3_)_3_]	98.99	97.82		98.71	99.27	5.44
	Karstedt	98.30	45.89		35.15	34.05	34.12
**[S_222_][NTf_2_] 4**	K_2_PtCl_4_	86.03	85.17		84.93	86.35	84.41
	K_2_PtCl_6_	87.80	83.62		86.47	85.03	84.57
	Pt(PPh_2_)_2_Cl_2_	85.52	85.43		85.66	85.77	86.30
	Pt(PPh_3_)_4_	90.00	90.00		85.00	72.00	67.00
	[RhCl(PPh_3_)_3_]	94.00	97.00		89.00	62.00	7.00
	Karstedt	86.03	85.17		0.00	0.00	0.00
**[AllPy][NTf_2_] 5**	K_2_PtCl_4_	58.00	47.00	14.00		3.00	1.00
	K_2_PtCl_6_	91.00	66.00	31.00		10.00	1.00
	Pt(PPh_2_)_2_Cl_2_	47.00	32.00	23.00		14.00	9.00
	Pt(PPh_3_)_4_	39.00	22.00	13.00		3.00	1.00
	[RhCl(PPh_3_)_3_]	88.00	0.00	0.00		x	x
	Karstedt	97.15	93.70	57.58		6.08	0.00
**[diAllMIM][NTf_2_] 6**	K_2_PtCl_4_	1.00	0.00	0.00		x	x
	K_2_PtCl_6_	0.00	0.00	0.00		x	x
	Pt(PPh_2_)_2_Cl_2_	15.00	11.00	8.00		9.00	2.00
	Pt(PPh_3_)_4_	3.00	3.00	3.00		0.00	0.00
	[RhCl(PPh_3_)_3_]	0.00	0.00	0.00		0.00	0.00
	Karstedt	16.00	9.00	6.00		2.00	0.00
**[AlldiMIM][NTf_2_] 7**	K_2_PtCl_4_	10.00	8.00	2.00		0.00	0.00
	K_2_PtCl_6_	2.00	5.00	3.00		0.00	0.00
	Pt(PPh_2_)_2_Cl_2_	35.00	27.00	20.00		14.00	11.00
	Pt(PPh_3_)_4_	30.00	25.00	19.00		8.00	0.00
	[RhCl(PPh_3_)_3_]	78.00	18.00	2.00		0.00	0.00
	Karstedt	30.00	15.00	8.00		3.00	0.00

^1^ Yields color code: green > 90%; blue 70–90%; orange 50–70%; yellow 30–50%; red 0–30%; [P_44414_][NTf_2_] tributyltetradecylphosphonium bis(trifluoromethylsulfonyl)imide; [BuPy][NTf_2_] 1-butylpyridinium bis(trifluoromethylsulfonyl)imide; [BMMIM][NTf_2_] 1-butyl-2,3-dimethylimidazolium bis(trifluoromethylsulfonyl)imide; [S_222_][NTf_2_] triethylsulfonium bis(trifluoromethylsulfonyl)imide; [AllPy][NTf_2_] 1-allylpyridinium bis(trifluoromethylsulfonyl)imide; [diAllMIM][NTf_2_] 1,3-diallyl-2-methylimiidazolium bis(trifluoromethylsulfonyl)imide; [AlldiMIM][NTf_2_] 1-allyl-2,3-dimethylimidazolium bis(trifluoromethylsulfonyl)imide.

## 2. New Table 1

**Table 1:** Yields of hydrosilylation reaction in subsequent cycles for biphasic systems of ionic liquids and catalysts used.

Ionic Liquid	Catalyst	Yields in Subsequent Cycles (%) ^1^				
		1	2	3	4	5
**[P_44414_][NTf_2_] 1**	K_2_PtCl_4_	98.34	11.73	6.56	4.53	3.57
	K_2_PtCl_6_	70.26	58.65	13.09	10.87	9.56
	Pt(PPh_3_)_2_Cl_2_	100.00	100.00	100.00	95.25	87.74
	Pt(PPh_3_)_4_	99.82	99.22	99.13	22.40	4.77
	[RhCl(PPh_3_)_3_]	98.45	99.60	98.68	98.71	98.71
	Karstedt	99.27	99.12	98.95	99.19	55.41
**[BuPy][NTf_2_] 2**	K_2_PtCl_4_	85.68	2.25	0.39	0.75	3.92
	K_2_PtCl_6_	86.05	3.42	5.47	10.74	13.58
	Pt(PPh_3_)_2_Cl_2_	84.22	2.30	5.23	5.68	6.38
	Pt(PPh_3_)_4_	16.12	14.79	13.14	11.46	11.52
	[RhCl(PPh_3_)_3_]	100.00	12.22	9.56	2.58	8.91
	Karstedt	100.00	80.09	16.02	17.37	9.44
**[BMMIM][NTf_2_] 3**	K_2_PtCl_4_	60.26	55.68	15.15	16.75	17.40
	K_2_PtCl_6_	29.35	28.69	27.45	30.26	33.48
	Pt(PPh_3_)_2_Cl_2_	100.00	100.00	86.24	85.84	59.07
	Pt(PPh_3_)_4_	86.39	85.46	84.81	84.52	85.18
	[RhCl(PPh_3_)_3_]	98.99	97.82	98.71	99.27	5.44
	Karstedt	98.30	45.89	35.15	34.05	34.12
**[S_222_][NTf_2_] 4**	K_2_PtCl_4_	86.03	85.17	84.93	86.35	84.41
	K_2_PtCl_6_	87.80	83.62	86.47	85.03	84.57
	Pt(PPh_3_)_2_Cl_2_	85.52	85.43	85.66	85.77	86.30
	Pt(PPh_3_)_4_	90.00	90.00	85.00	72.00	67.00
	[RhCl(PPh_3_)_3_]	94.00	97.00	89.00	62.00	7.00
	Karstedt	86.03	85.17	0.00	0.00	0.00
**[AllPy][NTf2] 5**	K_2_PtCl_4_	58.00	47.00	14.00	3.00	1.00
	K_2_PtCl_6_	91.00	66.00	31.00	10.00	1.00
	Pt(PPh_2_)_2_Cl_2_	47.00	32.00	23.00	14.00	9.00
	Pt(PPh_3_)_4_	39.00	22.00	13.00	3.00	1.00
	[RhCl(PPh_3_)_3_]	88.00	0.00	0.00	x	x
	Karstedt	97.15	93.70	57.58	6.08	0.00
**[diAllMIM][NTf_2_] 6**	K_2_PtCl_4_	1.00	0.00	0.00	x	x
	K_2_PtCl_6_	0.00	0.00	0.00	x	x
	Pt(PPh_3_)_2_Cl_2_	15.00	11.00	8.00	9.00	2.00
	Pt(PPh_3_)_4_	3.00	3.00	3.00	0.00	0.00
	[RhCl(PPh_3_)_3_]	0.00	0.00	0.00	0.00	0.00
	Karstedt	16.00	9.00	6.00	2.00	0.00
**[AlldiMIM][NTf_2_] 7**	K_2_PtCl_4_	10.00	8.00	2.00	0.00	0.00
	K_2_PtCl_6_	2.00	5.00	3.00	0.00	0.00
	Pt(PPh_3_)_2_Cl_2_	35.00	27.00	20.00	14.00	11.00
	Pt(PPh_3_)_4_	30.00	25.00	19.00	8.00	0.00
	[RhCl(PPh_3_)_3_]	78.00	18.00	2.00	0.00	0.00
	Karstedt	30.00	15.00	8.00	3.00	0.00

^1^ Yields color code: green >90%; blue 70–90%; orange 50–70%; yellow 30–50%; red 0–30%; [P_44414_][NTf_2_] tributyltetradecylphosphonium bis(trifluoromethylsulfonyl)imide; [BuPy][NTf_2_] 1-butylpyridinium bis(trifluoromethylsulfonyl)imide; [BMMIM][NTf_2_] 1-butyl-2,3-dimethylimidazolium bis(trifluoromethylsulfonyl)imide; [S_222_][NTf_2_] triethylsulfonium bis(trifluoromethylsulfonyl)imide; [AllPy][NTf_2_] 1-allylpyridinium bis(trifluoromethylsulfonyl)imide; [diAllMIM][NTf_2_] 1,3-diallyl-2-methylimiidazolium bis(trifluoromethylsulfonyl)imide; [AlldiMIM][NTf_2_] 1-allyl-2,3-dimethylimidazolium bis(trifluoromethylsulfonyl)imide.

## 3. Former Figure 3

**Figure 3:**
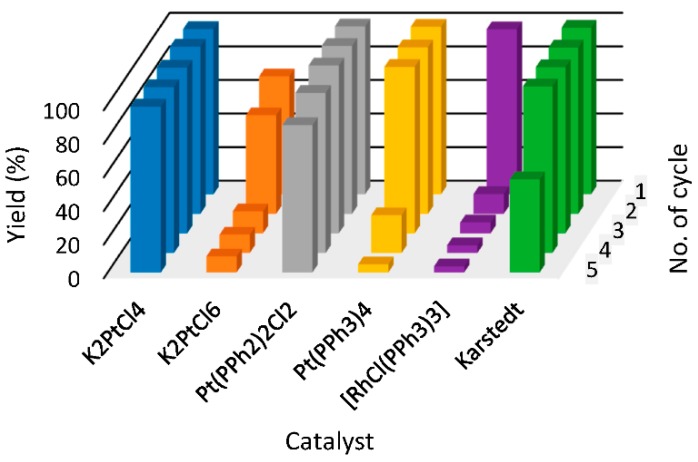
The yields of hydrosilylation reaction carried out in [P_44414_][NTf_2_] (**1**) (tributyltetradecylphosphonium bis(trifluoromethylsulfonyl)imide) in subsequent cycles. Colors refer to different catalysts used in hydrosilylation reaction.

## 4. New Figure 3

**Figure 3:**
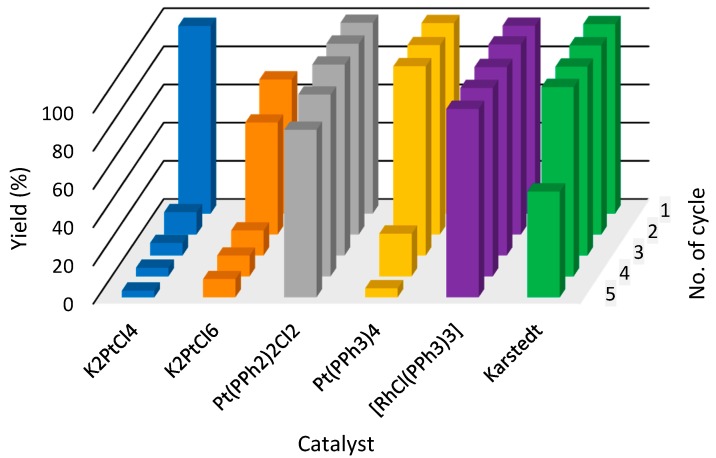
The yields of hydrosilylation reaction carried out in [P_44414_][NTf_2_] (**1**) (tributyltetradecylphosphonium bis(trifluoromethylsulfonyl)imide) in subsequent cycles. Colors refer to different catalysts used in hydrosilylation reaction.

Moreover, there are four mistakes in the article text:

1. On page 3, lines 32–34, the sentence “[P_44414_][NTf_2_] (**1**) IL system shows satisfying yields close to 100% throughout the whole five reaction cycles for catalysts such as K_2_PtCl_4_, Pt(PPh_2_)_2_Cl_2_ and Karstedt catalyst (100% yield up to fourth cycle)” should be replaced with “[P_44414_][NTf_2_] (**1**) IL system shows satisfying yields close to 100% throughout the whole five reaction cycles for catalysts such as [RhCl(PPh_3_)_3_], Pt(PPh_3_)_2_Cl_2_ and Karstedt catalyst (100% yield up to fourth cycle)”.
2. On page 3, lines 38 and 39, the sentence “The least effective catalyst for IL (**1**) is rhodium catalyst for which a major drop in yield was observed after the first reaction cycle” should be replaced with “The least effective catalyst for IL (**1**) is platinum catalyst, K_2_PtCl_4_, for which a major drop in yield was observed after the first reaction cycle”.
3. On page 7, lines 10–12, the sentence “[the] most efficient IL systems for hydrosilylation reaction were [P_44414_][[NTf_2_] (**1**)/K_2_PtCl_4_ and [P_44414_][NTf_2_] (**1**)/Pt(PPh_2_)_2_ for which yields after a fifth cycle were maintained at a level of more than 80%.” should be replaced with “The most efficient IL systems for hydrosilylation reaction were [P_44414_][[NTf_2_] (**1**)/[RhCl(PPh_3_)_3_] and [P_44414_][NTf_2_] (**1**)/Pt(PPh_3_)_4_, for which yields after a fifth cycle were maintained at a level of more than 80%”.
4. On page 7, lines 15 and 16, the statement “which in four out of seven tested IL systems shows [an] immediate drop of the catalyst activity when being recycled for the first time.” should be replaced with “which in three out of seven tested IL systems shows an immediate drop of the catalyst activity when being recycled for the first time”.

The authors would like to apologize for any inconvenience caused to the readers by these changes.
